# Supplementary material for: Patterns of Intron Gain and Loss in Fungi
Source: PLoS Biol. 2004 Nov 30;2(12):e422. doi: 10.1371/journal.pbio.0020422 (PMC532390; doi:10.1371/journal.pbio.0020422)
Supplement: Table S1 — Also available at http://genes.mit.edu/NielsenEtAl/. (4.3 MB ZIP). [file pbio.0020422.st001.zip › NielsenEtAl/html/683.html]

AN7705.1.NCU00879.1.MG10588.1.FG02812.1


```
 CLUSTAL W (1.82) Multiple Sequence Alignments - Introns Inserted


Sequence 1: NCU00879.1	352 aa
Sequence 2: FG02812.1	349 aa
Sequence 3: MG10588.1	351 aa
Sequence 4: AN7705.1	340 aa
Alignment Length: 365 aa
Number Identitical Residues: 144 aa
Alignment Score (without introns) 8011


MG10588.1 	MATEA~PRQYVPLTCHGHSRPVPHINFSSTFES------ENYFMISACKD1GNPMLRQGQ
NCU00879.1	MTEPA~RRQTVPLTCHGHSRPVTHLSFSPLEKE------DSYYMISSCKD1GNPMLRDGQ
FG02812.1 	-MASE~PRQYHPLTCHGHSRPVPHIAFSAIEKDTDKDKNDVYYMISACKD1GNPMLRNGI
AN7705.1  	-MASD1IPKVVPLTCHGHSRPVPHISFSSTVED------DQYYLVSACKD1NNPMLRDGI
          	        :  ***********.*: **.  :.      : *:::*:*** .*****:* 

MG10588.1 	TGDW~IGTFIGHKGAVWQAKLSPDMSNAATASADFSC2KIWNSHTGEVLFTLKHEHIVRA
NCU00879.1	TGDW~IGTFLGHKGATWQARLSPDASTAATSSADFSA~KIWDTHTGELLYVLKHDHIVRA
FG02812.1 	TGDW2IGTLIGHKGAVWQARLSPDAHHAATASADFTA2KIWDTYTGELLHTLQHDHIVRA
AN7705.1  	TGDW2IGTFLGHKGAVWQARLSTDANIAATAAADFSA2KVWDTHTGQCLHTLQHSHIVRA
          	**** ***::*****.***:**.*   ***::***:. *:*:::**: *..*:*.*****

MG10588.1 	IAYPPNTS-DMVATGGFEKKLRLWDLQAA-SKKLAET-----TEEVAVESSEAHEIGAGT
NCU00879.1	IAYPYENS-GLLATGGYEKKLRIFDLADQ-RPATSPSGT--SPEPVTIDASKAFEIGEGV
FG02812.1 	IAYPPDNS-DLIATGGMEKKLRIFDLSEL-PVAGSPT---------TINASTGFEIGEGV
AN7705.1  	VAFPLQPSPQVLATGGYEKKLRIFDLTRSGSNSSSPTASSAPPAEDGTSSATSYEIGPGA
          	:*:* : *. ::**** *****::**   .    : :.:::..     .:: ..*** *.

MG10588.1 	HTDPIKAIVWAPDPTKIITASG-KTLRWFDVPTKSLVKTLVLDDEIKSCESSQLDPQFAD
NCU00879.1	HKDIIKFIVWARDPNVIITASG-DTLRWFDLPSRRCVREAKLEGEIKSCELVSLAPSHSA
FG02812.1 	HTGSIKFICWTQDPNIVVTASD-KTIRWLDLPSRSCIRHEVLDGDIKSCEMVSLAPQYAS
AN7705.1  	HGGTIKSIVWNRDYNILTTAADDRKIRWWDLRSRHPVVEYPVEGNIGSCELNTLATR---
          	* . ** * *  * . : **:.. .:** *: ::  :    ::.:* ***   * .    

MG10588.1 	SSDINGGSPVLAVAAGKTVVFFGGRQMNEELKRIKLTHGIASVALDLKQRKFAVGEEPGT
NCU00879.1	PTDIGGGLPVLAVAAGKTAYFWGGSRAEDELKRITLPHGIASVGLDLKGRKFVVGEEPGT
FG02812.1 	PSDIGGGKPVLAVAAGKTAYFWGGVQAMDELKRISLTYTIASVSLDLKGRKLVVGEEPGT
AN7705.1  	PNDTG----ILSVAAGKSVYLFDGHTPGRLLKKIDFGYEVASAAVNNDTARLVTGSADDT
          	..* .    :*:*****:. ::.*      **:* : : :**..:: .  ::..*.  .*

MG10588.1 	WVRVYSYEDEKEID1VHKGHHGPVWSIAFSPDGKLYATASEDGTIKLWKNCEAFYGLWRG
NCU00879.1	WARVYTWDEGQEID~VHKGHHGPIWSIAFSPDGNLYATGSEDGTIKMWKNCDGYYGLWRG
FG02812.1 	WAKVIRYDDGTEVD~THKGHHGPIWSIAFSPDGKLYATGSEDGTIKLWKNCEGYYGLWRG
AN7705.1  	WARVYDLHTDDELE1VQKGHHGPIWSVSFSPDGKLYGTGSEDGTIKLWKACREPYGLWR-
          	*.:*   .   *:: .:******:**::*****:**.*.*******:** *   ***** 

MG10588.1 	GANGTEKSSD
NCU00879.1	GVSGSGTD--
FG02812.1 	GAEKAAE---
AN7705.1  	----------
          	
```
